# Short-Term Effects of Manual Therapy Combined with Functional Magnetic Stimulation in Individuals with Lumbar Disk Herniation with Radiculopathy: A Randomized Clinical Trial

**DOI:** 10.3390/medicina62020249

**Published:** 2026-01-24

**Authors:** Dimitrios Lytras, Paris Iakovidis, Konstantinos Kasimis, Vasileios Georgoulas, Ioannis Algiounidis, Georgia Maria Kamparoudi, Georgios Tsigaras, Georgia Tarfali, Georgia Vergidou, Nikolaos Sidiropoulos, Eleftheria Zerva, Ilias Kallistratos

**Affiliations:** 1 Laboratory of Biomechanics & Ergonomics, Department of Physiotherapy, Faculty of Health Sciences, International Hellenic University, Sindos, 57400 Thessaloniki, Greece; piakov@ihu.gr (P.I.);; 2 G. Papanikolaou Hospital, 57010 Thessaloniki, Greece; 3 Department of Physiotherapy, Faculty of Health Sciences, International Hellenic University, Sindos, 57400 Thessaloniki, Greece; gkamparoudi@ihu.gr (G.M.K.);; 4 Laboratory of Basic and Applied Research in Physiotherapeutic Rehabilitation, Department of Physiotherapy, Faculty of Health Sciences, International Hellenic University, Sindos, 57400 Thessaloniki, Greece; tsigarasg@ihu.gr (G.T.); elikall@ihu.gr (I.K.); 5 School of Health Sciences, Queen Margaret University, Edinburgh EH21 6UU, UK

**Keywords:** lumbar disk herniation, radiculopathy, manual therapy, functional magnetic stimulation, neuropathic pain, spinal mobilization, physiotherapy

## Abstract

*Background and Objectives*: Lumbar disk herniation with radiculopathy (LDHR) is a prevalent neuromusculoskeletal condition characterized by nociceptive and neuropathic pain components. Manual therapy (MT) is commonly used in its management, whereas Functional Magnetic Stimulation (FMS) represents an emerging modality with limited evidence in radiculopathy. The aim of this study was to examine the short-term effects of combining MT with FMS compared with MT alone on pain intensity, neuropathic pain features, neural mechanosensitivity, and functional disability in individuals with chronic LDHR. *Materials and Methods*: Forty adults with MRI-confirmed unilateral LDHR were randomly allocated to an MT + FMS group or an MT-only group. Both groups received ten treatment sessions over three weeks. Outcomes included lumbar and leg pain intensity (NPRS), functional disability (RMDQ), neuropathic pain symptoms (S-LANSS), and straight leg raise (SLR) range of motion. Measurements were obtained at baseline and at week 3. Group and time effects were examined using a two-way mixed ANOVA with significance set at *p* < 0.05. *Results*: Significant group × time interactions were observed for all outcomes (*p* < 0.01), indicating greater improvements in the MT + FMS group. Reductions in lumbar and leg pain, disability, and S-LANSS scores exceeded established MCID thresholds, while SLR gains surpassed published MDC values, reflecting both statistical and clinical relevance. Only the MT + FMS group improved below the neuropathic pain diagnostic cutoff (S-LANSS < 12). *Conclusions*: The findings of this trial suggest that incorporating FMS into a manual therapy program may provide additional short-term clinical benefits for individuals with chronic LDHR. Further research with larger samples, longer follow-up periods, and mechanistic assessments is needed to confirm these preliminary results and to better understand the underlying mechanisms.

## 1. Introduction

Low back pain constitutes one of the leading causes of disability worldwide, exerting a substantial burden on individuals, healthcare systems, and society as a whole [1]. Among the etiologies of low back pain, lumbar disk herniation with radiculopathy (LDHR) represents one of the most clinically distinct and well-characterized entities [2]. The diagnosis is typically based on a combination of symptoms and clinical signs indicating irritation or compression of the lumbar spinal nerve roots, such as radicular (sciatic) pain, neurological deficits, and imaging findings that correlate with the clinical presentation [3]. Lumbar disk herniation is defined as the localized displacement of intervertebral disk material beyond the normal margins of the disk space, often resulting in nerve root compression and lower limb symptoms [3,4].

Lumbar disk herniation is a common degenerative disorder of the spine [5], while its symptomatic form with radiculopathy (LDHR) has a point prevalence of approximately 5% in adults—particularly among individuals aged 30–50 years—and is associated with high levels of pain, functional impairment, and increased healthcare utilization [2]. The most frequently affected levels are L4–L5 and L5–S1 [3], and factors such as smoking, elevated body mass index, and prolonged mechanical loading of the lumbar spine have been linked to a higher risk of developing LDHR [2]. In parallel, only a small proportion of patients with LDHR and persistent symptoms ultimately undergo surgical management [5].

Recent international clinical guidelines indicate that, in the absence of red-flag symptoms, conservative treatment represents the first-line approach, as the majority of patients show improvement without surgical intervention [5]. Nevertheless, clinical management remains challenging, and there is a continued need for interventions that provide faster and more meaningful pain reduction, improvement of neuropathic symptoms, and restoration of functional capacity. The search for more effective or combination-based conservative strategies, therefore, remains an active area of both clinical and research interest.

Moreover, although a substantial proportion of patients with LDHR show improvement within the first 4–6 weeks of conservative management, a considerable subset continues to experience persistent or chronic symptoms, leading to significant neuropathic pain and functional limitation [3]. The chronic form of the condition (chronic LDHR) carries particular clinical importance, as it is associated with increased disability and often requires targeted and more intensive conservative interventions [3,6,7].

Manual therapy has been shown to provide meaningful benefits for patients with LDHR, contributing to pain reduction and improved functional outcomes [7,8]. Specific techniques, such as spinal mobilization with leg movement (SMWLM) as described by Mulligan and neurodynamic mobilization using sliders and tensioners, have been documented as effective in improving symptoms, particularly in chronic cases [6,7]. The analgesic mechanisms of these techniques are proposed to include reduced mechanical loading on the irritated neural structures, enhanced neurodynamic sliding, and modulation of neural sensitivity [9,10].

Another conservative approach for the management of LDHR is magnetic stimulation. Preliminary clinical evidence suggests that its application may reduce radicular pain, with some studies also reporting improvements in functional capacity [11]. In contrast to pulsed electromagnetic fields, which operate at low-intensity levels—typically below 100 mT—and primarily exert cellular and anti-inflammatory effects without the ability to induce neuromuscular activation [12,13], Functional Magnetic Stimulation (FMS) delivers much higher-intensity magnetic fields (0.5–3 Tesla) capable of inducing electric currents in motor axons and producing actual muscle contractions [14,15]. Moreover, recent findings in patients with sciatica have shown that incorporating FMS into a physiotherapy program significantly accelerates improvements in lumbar mobility and SLR, with early changes observed within the first few days of application [15].

Although manual therapy and functional magnetic stimulation have been studied as separate conservative approaches for LDHR, the theoretical rationale for combining these modalities has received limited attention. From a mechanistic perspective, manual and neurodynamic techniques primarily deliver mechanical afferent input to neural tissues, potentially reducing mechanosensitivity and improving neural mobility [7,9,10], while FMS provides frequency-modulated electromagnetic stimulation capable of influencing neuromuscular and nociceptive processing through peripheral and potentially central neuromodulatory pathways [14,15]. It may therefore be hypothesized that the concurrent application of mechanical mobilization and electromagnetic stimulation could produce additive or complementary effects by engaging distinct but interacting mechanisms involved in pain modulation, neural sensitivity, and functional recovery. However, this proposed interaction should be regarded as a hypothesis rather than a confirmed synergistic mechanism.

In the context of LDHR, this hypothesized interaction may be particularly relevant, given that radicular pain and functional limitation are strongly associated with increased neural mechanosensitivity, impaired intraneural mobility, and heightened nociceptive input. Neurodynamic mobilization may improve tolerance to neural loading and reduce mechanically evoked symptoms, while the concurrent application of FMS may modulate peripheral nociceptive gain and facilitate descending inhibitory mechanisms. The simultaneous delivery of mechanical and electromagnetic stimulation during movement may therefore contribute to improved neural responsiveness and symptom modulation in patients with chronic lumbar radiculopathy. However, these proposed effects remain hypothetical and require further mechanistic investigation.

Despite growing interest in combining manual therapy and functional magnetic stimulation, several uncertainties remain regarding their mechanisms and clinical effects. Individual responses to magnetic stimulation may vary depending on factors such as neural sensitivity, symptom chronicity, and central pain modulation [16], while manual therapy interventions are known to involve non-specific effects related to patient expectations, therapeutic context, and clinician–patient interaction [17]. Acknowledging these sources of variability underscores the importance of controlled clinical investigations to clarify whether combining these modalities provides benefits beyond those attributable to contextual or placebo-related influences alone.

The aim of the present clinical study was to evaluate the effect of concurrently integrating FMS within a manual therapy protocol based on neural tissue mobilization techniques in patients with chronic LDHR. The study was designed under the hypothesis that the concurrent and combined application of neurodynamic mobilization techniques and FMS, integrating their analgesic mechanisms, would yield superior outcomes in pain reduction, disability, and improvement of neuropathic symptoms compared with manual therapy alone.

## 2. Materials and Methods

### 2.1. Study Design

This study was conducted under the supervision of the Department of Physiotherapy at the International Hellenic University in Thessaloniki. Its implementation followed the CONSORT guidelines for randomized clinical trials and was designed as a two-arm, randomized, controlled clinical trial with assessor blinding and a 1:1 allocation ratio between the two groups. The research protocol was approved by the Ethics Committee of the Department of Physiotherapy at the International Hellenic University (approval number: EC-16 2025), and the study was registered on ClinicalTrials.gov (ID: NCT07234071). All participants provided written informed consent prior to randomization. The CONSORT checklist is provided as Supplementary Materials.

### 2.2. Sampling, Randomization, and Blinding

Participants were recruited from two outpatient clinics located in Thessaloniki, Greece. All potential participants had previously been evaluated by an orthopedic physician, received a clinical diagnosis of lumbar disk herniation with radiculopathy confirmed by MRI, and had a referral for physiotherapy. Initially, each individual who expressed interest in taking part in the study was informed by members of the research team about the study’s purpose and procedures. Subsequently, an orthopedic physician with 10 years of clinical experience in musculoskeletal disorders conducted the eligibility assessment for each potential participant.

After providing written informed consent and completing the baseline assessment, participants received a unique identification code and were randomly allocated in a 1:1 ratio to either the manual therapy + FMS group or the manual therapy–only group. Randomization was performed by an independent researcher using the Research Randomizer (Version 4) [18], with stratification by outpatient clinic and random permuted blocks of variable size. Allocation concealment was ensured using sequentially numbered, opaque, sealed envelopes, which were opened in order of enrollment.

Due to the nature of the interventions, blinding of therapists and participants was not feasible. However, all pre- and post-intervention measurements were performed by an independent assessor who was blinded to group allocation.

### 2.3. Participants

The inclusion criteria were: (1) age 18–64 years, (2) clinically diagnosed unilateral LDHR by an orthopedic physician, confirmed by MRI showing disk herniation at the L4/L5 and/or L5/S1 levels, (3) referral for physiotherapy, (4) pain distributed along the sciatic nerve (leg-dominant symptoms) and a positive Straight Leg Raise test, (5) symptom duration of ≥12 weeks, and (6) provision of written informed consent.

Exclusion criteria were: (1) presence of red flags (cauda equina syndrome, infection, or malignancy), (2) bilateral radiculopathy, (3) previous lumbar spine surgery or scheduled surgical intervention during the study period, (4) receipt of interventional treatments (e.g., epidural injections) within the previous six weeks, (5) severe neurological, metabolic, or inflammatory rheumatologic disease unrelated to the current condition, (6) contraindications to magnetic stimulation such as a pacemaker, implanted neurostimulator, cochlear implant, or significant metallic implant in the treatment area, and (7) pregnancy or breastfeeding.

### 2.4. Measurements

The following measurements were conducted at baseline and after the third week. All outcomes were considered primary and were performed by the same independent assessor, who was blinded to group allocation.

#### 2.4.1. Pain Intensity—Numeric Pain Rating Scale (NPRS)

Pain intensity was assessed using the 11-point Numeric Pain Rating Scale (NPRS), where 0 corresponds to “no pain” and 10 to “the worst pain imaginable,” with higher scores indicating greater pain intensity [19]. For the purposes of the present study, two distinct pain measurements were recorded: lumbar pain (NPRS–Lumbar pain) and radicular/sciatic pain in the lower limb (NPRS–Leg pain), in accordance with contemporary literature on patients with LDHR [6,20]. Participants were asked to rate the intensity of their lumbar and lower-limb pain over the previous 24 h. Since no established MCID values exist specifically for patients with lumbar radiculopathy, the clinical significance of changes was evaluated using the most well-supported values reported in the literature. For lumbar pain (NPRS–Lumbar pain), an MCID value of 2.5 points was adopted, as proposed for chronic low back pain by Ostelo and de Vet [21]. For radicular leg pain (NPRS–Leg pain), an MCID of 2 points was used, which is widely accepted as a threshold of clinical importance for chronic pain and has been documented in related clinical trials [7,22].

#### 2.4.2. Functional Disability—Roland–Morris Disability Questionnaire (RMDQ)

Functional disability related to low back pain was assessed using the Roland–Morris Disability Questionnaire (RMDQ). The questionnaire consists of 24 statements describing limitations in daily activities due to low back pain. Each item is answered with “Yes” or “No,” and the total score reflects the number of statements marked “Yes,” ranging from 0 (no disability) to 24 (maximum disability), with higher scores indicating greater functional limitation [23]. The RMDQ is considered one of the most well-validated instruments for assessing disability in individuals with low back pain and has been used in several clinical trials involving patients with LDHR and unilateral radiculopathy [6,7]. For the purposes of the present study, the Greek version of the questionnaire was used, for which internal consistency has been reported as Cronbach’s α = 0.885 [24]. To determine the clinical significance of changes in the RMDQ, an MCID of ≥5 points was adopted. This choice is supported by the findings of Lauridsen et al. [25], who identified a 5-point change as the optimal cut-off for meaningful improvement in patients with low back pain and lower-limb pain (leg pain ± LBP). This value also falls within the MCID range reported by Ostelo and de Vet [21] for the RMDQ (approximately 2–7/8 points depending on baseline disability) and exceeds the minimum proposed threshold of ≥3.5 points for clinically important improvement in low back pain.

#### 2.4.3. Neuropathic Pain Features—Self-Report Leeds Assessment of Neuropathic Symptoms and Signs (S-LANSS)

Neuropathic pain features were assessed using the Self-report Leeds Assessment of Neuropathic Symptoms and Signs (S-LANSS), a seven-item questionnaire that yields a total score ranging from 0 to 24, with values ≥ 12 considered indicative of a neuropathic pain mechanism [26]. The instrument was developed for the identification of neuropathic pain and demonstrates satisfactory diagnostic accuracy in chronic pain conditions, with sensitivity reported at approximately 74–78% and overall correct classification at 75–80% [26]. For the purposes of the present study, the Greek version of the S-LANSS was used. This version has been translated, culturally adapted, and validated, demonstrating sensitivity of 86.21%, specificity of 95.24%, Cronbach’s α = 0.67, and high test–retest reliability (r = 0.964) for the ≥12 threshold [27]. Since no established MCID or MDC values exist for the S-LANSS total score in patients with LDHR, interpretation of the results was based on changes in the total score and on the proportion of participants exceeding the ≥12 threshold, in accordance with previous clinical trials in lumbar radiculopathy [7].

#### 2.4.4. Mechanical Sensitivity of the Sciatic Nerve—Straight Leg Raise (SLR) with Goniometer

Mechanical sensitivity of the sciatic nerve and lumbosacral nerve roots was assessed using the passive Straight Leg Raise (SLR) test, performed with a universal goniometer (MSD Europe, Watermael-Boitsfort, Belgium). Participants were positioned in the supine position with the pelvis stabilized, and the examiner passively raised the affected lower limb with the knee fully extended. During the assessment, the angle at which radicular pain occurred (pain-limited SLR angle) was recorded and used as the primary measure of mechanical sensitivity. SLR values are expressed in degrees (°), with higher angles indicating lower mechanical sensitivity and improved neurodynamic behavior. The passive SLR test has been documented as a reliable clinical measure both for diagnosing disk herniation and for evaluating mechanical sensitivity of neural tissues, demonstrating high sensitivity (91%) but low specificity (26%) [7,28]. Additionally, published values exist regarding detectable change thresholds. Neto et al. [29] reported that changes of approximately 7–8° can be considered minimally detectable, whereas Dixon and Keating [30] indicated that changes greater than 16° are required to reflect clinically meaningful differences between measurements.

### 2.5. Experimental Protocols

#### 2.5.1. Manual Therapy

Participants in the manual therapy group followed a standardized manual therapy protocol applied every other day for three consecutive weeks (10 sessions in total), with each session lasting 25 min. Each session began with 10 min of preparatory massage to the lumbar region, gluteal musculature, and posterior thigh on the symptomatic side, in order to reduce soft-tissue sensitivity and prepare the limb for neurodynamic mobilization. Following massage preparation, participants received a standardized sequence of neurodynamic techniques, beginning with SMWLM according to Mulligan [6,31], immediately followed by neurodynamic mobilization. The neurodynamic component of the protocol was based on mobilization procedures previously implemented in similar clinical trials [7,32]. A detailed description of the manual therapy protocol is provided in Table 1. Treatment fidelity was ensured through structured therapist training and systematic per-session documentation. All care providers delivering the interventions were licensed physiotherapists with prior clinical experience in manual therapy and neurodynamic techniques, and received protocol-specific training before study initiation. For each session, the lumbar level and direction of the applied glide, the number of sets and repetitions, the type of neurodynamic technique used (sliders or tensioners), and the immediate symptom response during execution were recorded. In addition, at every visit, the angle of symptom onset and the maximum tolerable angle during the SLR test were documented. Sessions were modified or temporarily discontinued when an increase in more than 2 points on the NPRS in radicular pain occurred and did not resolve by the end of the session, while treatment was terminated entirely in the presence of red flags or progressive neurological deficit. Any adverse events were systematically recorded at each session.

#### 2.5.2. Manual Therapy and FMS

**Figure 1:**
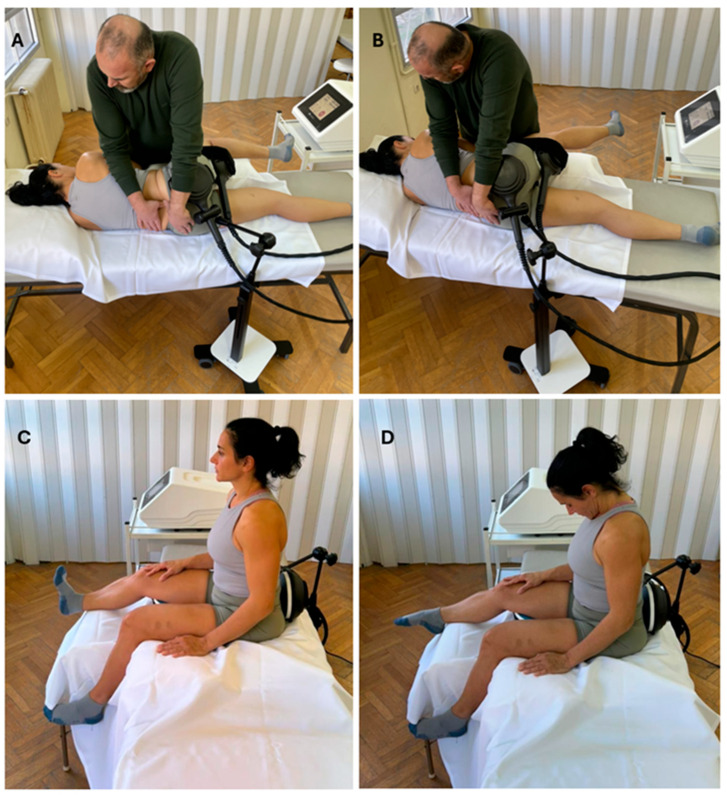
Application of Manual Therapy and Functional Magnetic Stimulation (FMS). (**A**,**B**) Concurrent application of FMS during spinal mobilization with limb movement (SMWLM). One coil is positioned over the sciatic nerve exit point in the gluteal region, and a second coil along the posterior thigh to provide continuous stimulation along the sciatic pathway during manual mobilization. (**C**,**D**) Repositioning of the coils for seated neurodynamic techniques (sliders and tensioners). One coil is placed over the lumbar area and the other below the ischial tuberosity, ensuring uninterrupted magnetic stimulation throughout the neurodynamic movements.

Participants in the manual therapy plus FMS group followed the same manual therapy protocol as the control group. Each session included a 10 min preparatory massage and the same sequence of neurodynamic techniques (SMWLM followed by sliders and tensioners). During the manual techniques, however, FMS was applied at the same time along the path of the sciatic nerve using a dual-coil magnetic stimulator (Tesla Stym, Iskra Medical, Ljubljana, Slovenia). Magnetic stimulation was delivered simultaneously through both applicators to provide continuous stimulation along the course of the sciatic nerve during movement. During SMWLM, one coil was placed over the sciatic nerve exit point in the gluteal area, positioned directly over the piriformis muscle, and the second coil was secured with a Velcro strap along the posterior thigh to follow the sciatic pathway (Figure 1A,B). For the seated neurodynamic techniques (sliders and tensioners), the coils were repositioned: one over the lumbar region and the other just below the ischial tuberosity, so that stimulation was maintained along the nerve during these movements (Figure 1C,D). Magnetic stimulation was applied using a preset frequency- and intensity-modulated program ranging from 3 to 50 Hz, consistent with the manufacturer’s clinical protocol for chronic sciatica. This protocol represents a preconfigured, condition-specific program that is routinely used in clinical practice for the management of chronic radicular pain. The present study was designed as a pragmatic clinical trial; therefore, stimulation parameters were not individually optimized or experimentally manipulated. Instead, functional magnetic stimulation was applied according to established manufacturer-recommended clinical settings in order to reflect real-world therapeutic application and to evaluate its additive clinical effect when combined with a standardized manual therapy program. Within the preset program, stimulation intensity was adjusted to an individually comfortable and clinically tolerable level that allowed smooth execution of the neurodynamic techniques without symptom exacerbation. The duration of FMS matched the 15 min duration of the neurodynamic techniques; FMS was not applied during the initial massage phase.

### 2.6. Sample Size Estimation

The required sample size was estimated using G*Power (version 3.1.9.7). Assuming a medium effect size (Cohen’s f = 0.25) [33], statistical power of 85%, and a two-tailed significance level of α = 0.05, the minimum total number of participants required was calculated to be 38. The choice of an effect size of 0.25 was informed by methodological recommendations and by evidence from randomized controlled trials in similar musculoskeletal and rehabilitation populations, where standardized target effect sizes typically range from 0.20 to 0.38 [34]. In the absence of prior trials examining the specific combination of manual therapy and FMS, this value was considered a conservative and methodologically appropriate estimate. Although a priori power analysis indicated that 38 participants were sufficient, we recruited 40 participants to allow for potential attrition and to ensure that the study maintained adequate statistical power in the event of dropouts.

### 2.7. Statistical Analysis

All statistical analyses were performed using IBM SPSS Statistics for Windows (Version 25.0; IBM Corp., Armonk, NY, USA) and R (Version 4.0.3). The distribution of continuous variables was assessed using the Shapiro–Wilk test together with visual inspection of Q–Q and P–P plots. Normally distributed variables are presented as mean ± standard deviation, whereas non-normally distributed variables are expressed as median and interquartile range. Categorical data are presented as frequencies and percentages. Between-group comparisons of baseline demographic and clinical characteristics were examined using independent-samples t-tests for normally distributed variables or the Mann–Whitney U test when assumptions of normality were violated. Comparisons of categorical variables were conducted using the Chi-square test.

To evaluate the effect of the intervention over time, a two-way mixed-design analysis of variance (ANOVA) was conducted, with one between-subject factor (“group”: manual therapy + FMS vs. manual therapy only) and one within-subject factor (“time”: baseline vs. week 3). The primary hypothesis test focused on the group × time interaction. When a statistically significant interaction was detected, simple main effects were examined using Bonferroni-adjusted pairwise t-tests. Effect sizes for ANOVA models were reported as partial eta-squared (η^2^p), with thresholds of 0.01, 0.06, and 0.14 denoting small, medium, and large effects, respectively [35]. The magnitude of between-group differences was quantified using Cohen’s d, interpreted according to the conventional thresholds described by Cohen [33]: d = 0.20 (small), d = 0.50 (medium), d = 0.80 (large). All randomized participants completed the intervention and both assessment time points; therefore, no data imputation was required. Statistical significance was set at α = 0.05 for all two-tailed tests. Ninety-five percent confidence intervals (95% CIs) were reported where applicable.

## 3. Results

**Figure 2:**
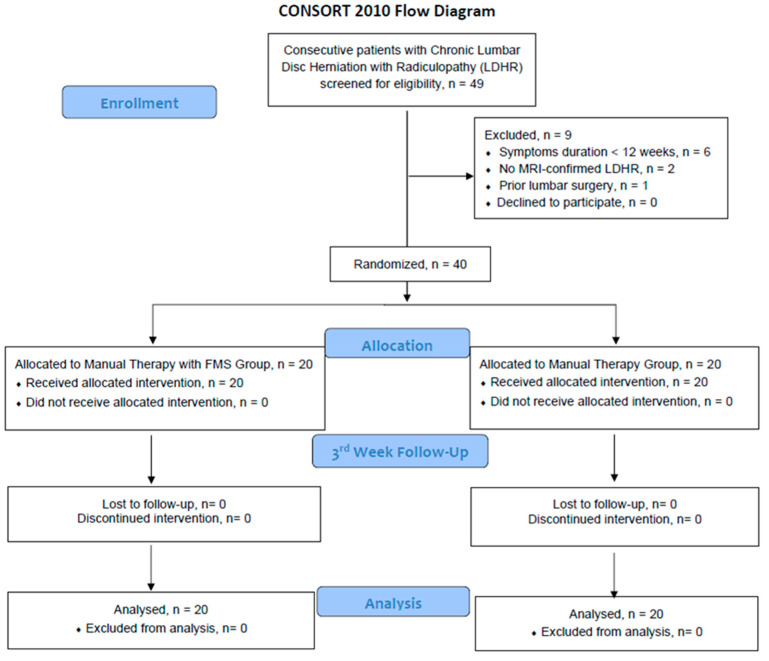
CONSORT Flow Diagram of the Study.

During the recruitment period, a total of 49 individuals expressed interest in participating in the study across the two outpatient clinics. Of these, 40 participants met all eligibility criteria and were enrolled in the trial (Figure 2). All enrolled participants (*n* = 40) completed the full 3-week intervention, attending all 10 treatment sessions, and were present at both assessment time points (baseline and week 3). When a session was missed for any reason, it was rescheduled within the same week—typically during the weekend—to maintain treatment consistency. No participant in either group reported any adverse event or undesired symptom related to the intervention throughout the study period.

The demographic and clinical baseline characteristics of the participants are shown in Table 2. Independent-samples *t*-tests and χ^2^ tests revealed no statistically significant differences between the two groups in any baseline variable, confirming that the groups were comparable prior to the intervention.

A significant group × time interaction was observed for lumbar pain intensity (NPRS–Lumbar), F(1,38) = 8.87, *p* = 0.005, η^2^p = 0.189 (Table 3), indicating that the two groups improved differently over time. A significant main effect of time was also detected, F(1,38) = 130.68, *p* < 0.001, η^2^p = 0.775, demonstrating that both groups showed substantial improvement from baseline to week 3. At baseline, lumbar pain levels did not differ significantly between groups (*p* = 0.324). By week 3, however, the FMS group reported significantly lower pain scores compared with the manual therapy group (mean difference = −1.20, *p* = 0.018, 95% CI: −2.18 to −0.22). The between-group effect size at week 3 was large (Cohen’s d = 0.78), favoring the FMS intervention. Within-group mean reductions were 3.75 points for the FMS group and 2.20 points for the manual therapy group. The improvement in the FMS group exceeded the MCID threshold for lumbar pain (2.5 points), whereas the improvement in the manual therapy group approached but did not exceed the MCID. Taken together, these findings indicate that combining FMS with manual therapy yields both statistically superior and clinically meaningful reductions in lumbar pain compared with manual therapy alone.

### 3.1. NPRS–Leg Pain Results

A highly significant group × time interaction was observed for radicular leg pain (NPRS–Leg), F(1,38) = 120.73, *p* < 0.001, η^2^p = 0.761 (Table 3), indicating that the magnitude of improvement differed markedly between the two interventions. A significant main effect of time was also present, F(1,38) = 893.78, *p* < 0.001, η^2^p = 0.959, demonstrating substantial overall improvement in both groups across the three-week intervention period. At baseline, no statistically significant differences were observed between groups (*p* = 0.267). By week 3, however, the FMS group exhibited significantly lower leg-pain intensity compared with the manual therapy group (mean difference = −1.45, *p* = 0.009, 95% CI: −2.52 to −0.38). The between-group effect size at week 3 was large (Cohen’s d ≈ 0.90), favoring the combined manual therapy + FMS intervention. Within-group reductions were −4.00 points for the FMS group (from 6.40 to 2.40) and −1.85 points for the manual therapy group (from 5.70 to 3.85). The reduction in the FMS group exceeded the established MCID threshold for radicular leg pain (2 points), while improvement in the manual therapy group fell below the MCID. Collectively, these findings indicate that the addition of FMS to manual therapy produced both statistically superior and clinically meaningful reductions in radicular leg pain compared with manual therapy alone.

### 3.2. RMDQ Results

A significant group × time interaction was found for functional disability (RMDQ), F(1,38) = 35.54, *p* < 0.001, η^2^p = 0.483 (Table 3), indicating that the pattern of improvement differed between groups across the intervention period. A strong main effect of time was also present, F(1,38) = 511.89, *p* < 0.001, η^2^p = 0.931, demonstrating substantial overall reductions in disability from baseline to week 3. At baseline, the two groups did not differ significantly in RMDQ scores (*p* = 0.547). By week 3, however, disability levels were significantly lower in the FMS group compared with the manual therapy group (mean difference = −3.35, *p* = 0.005, 95% CI: −5.64 to −1.06). The between-group effect size at week 3 was large (Cohen’s d ≈ 0.90), favoring the combined intervention. Within-group analyses showed an improvement of −9.95 points in the FMS group (from 14.00 to 4.05), exceeding the established MCID threshold of 5 points. In contrast, the manual therapy group improved by −5.80 points (from 13.20 to 7.40), which meets but does not exceed the recommended RMDQ MCID for meaningful clinical change. These findings indicate that although both groups improved, the addition of FMS resulted in substantially greater and clinically superior reductions in disability.

### 3.3. S-LANSS Results

A significant group × time interaction was identified for neuropathic pain features (S-LANSS), F(1,38) = 12.82, *p* = 0.001, η^2^p = 0.252 (Table 3), indicating that the magnitude of improvement differed between groups. A strong main effect of time was also present, F(1,38) = 848.47, *p* < 0.001, η^2^p = 0.957, demonstrating substantial overall reductions in neuropathic pain symptoms across the intervention period. At baseline, S-LANSS scores did not differ significantly between the two groups (*p* = 0.857). By week 3, however, the FMS group exhibited significantly lower neuropathic pain scores than the manual therapy group (mean difference = −2.45, *p* = 0.031, 95% CI: −4.66 to −0.24). The between-group effect size was large (Cohen’s d ≈ 0.85), favoring the combined intervention. Within-group analyses showed a marked reduction of −10.05 points in the FMS group (from 14.65 to 4.60) and a reduction of −7.85 points in the manual therapy group (from 14.90 to 7.05). Notably, only the FMS group reduced its mean score below the diagnostic threshold of ≥12, suggesting a clinically meaningful shift toward non-neuropathic pain classification at the group level. These findings indicate that although both groups improved, the addition of FMS produced greater and clinically more substantial reductions in neuropathic pain features.

### 3.4. SLR Results

A significant group × time interaction was found for SLR amplitude, F(1,38) = 104.12, *p* < 0.001, η^2^p = 0.733 (Table 3), indicating that the magnitude of improvement differed between groups. A strong main effect of time was also present, F(1,38) = 1394.13, *p* < 0.001, η^2^p = 0.973, demonstrating substantial overall increases in SLR range from baseline to week 3. At baseline, the two groups did not differ significantly in SLR values (*p* = 0.329). By week 3, however, participants receiving manual therapy combined with FMS achieved significantly greater SLR angles compared with those receiving manual therapy alone, with a mean between-group difference of 7.10°, *p* = 0.002, and a 95% CI ranging from 2.70° to 11.50°. The corresponding between-group effect size was large (Cohen’s d ≈ 1.10), indicating a clinically meaningful advantage of the combined intervention. Within-group improvement in the FMS group reached 20.85°, whereas the manual therapy group improved by 11.90°. Both changes exceeded the established Minimal Detectable Change thresholds of 7–8° [29], while the improvement in the FMS group also surpassed the ≥16° threshold considered reflective of clinically important change [30]. Overall, the addition of FMS resulted in markedly greater enhancement of neural mechanosensitivity, as reflected in the larger increase in SLR range compared with manual therapy alone.

## 4. Discussion

The present randomized controlled trial demonstrated that the concurrent application of FMS with manual therapy produced significantly greater improvements across all clinical outcomes compared with manual therapy alone in patients with chronic LDHR. Statistically significant group × time interactions were found for lumbar pain, leg pain, functional disability, neuropathic pain features, and sciatic nerve mechanosensitivity, indicating that the magnitude and trajectory of improvement differed substantially between the two interventions. In every outcome domain, the combined FMS + manual therapy group exhibited superior reductions in pain and disability, larger decreases in neuropathic symptomatology, and greater increases in SLR range. Importantly, these improvements were not only statistically significant but also exceeded established thresholds for clinical relevance (MCID and MDC), suggesting a clinically meaningful benefit associated with the integration of magnetic stimulation into manual therapy protocols. Overall, the findings support the possibility of an added clinical value of combining FMS with manual therapy, providing clinically meaningful improvements within a three-week treatment period. However, these results should be interpreted with caution until they are confirmed by larger and multicenter studies.

The present findings align closely with previous randomized trials demonstrating the effectiveness of manual therapy combined with neurodynamic techniques in patients with lumbar disk herniation. Studies by Plaza-Manzano et al. [7], Danazumi et al. [6], and Zainab et al. [32] consistently show that neurodynamic mobilization—particularly when integrated into a structured manual therapy program—produces meaningful reductions in radicular pain and improvements in functional outcomes. The additional contribution of FMS observed in the current trial is also compatible with the emerging evidence on electromagnetic stimulation in musculoskeletal disorders. Although the literature specifically examining FMS in sciatica is limited, recent clinical work by Radaković et al. [15] suggests that magnetic stimulation can reduce pain and enhance neuromuscular activation. Furthermore, evidence from studies on pulsed electromagnetic field therapies has demonstrated improvements in cellular metabolism, tissue healing, and analgesia, supporting the rationale for combining electromagnetic stimulation with mechanically based interventions [16]. Beyond the present findings, several multimodal RCTs—including those integrating physical agents with manual techniques—have shown that combined approaches tend to outperform monotherapies in reducing pain and restoring function [36,37,38,39]. Taken together, these data suggest that the simultaneous application of manual therapy and FMS is consistent with a broader body of evidence supporting multimodal treatment strategies in musculoskeletal rehabilitation.

The superior analgesic response observed in the FMS group is consistent with existing neurophysiological and biological evidence regarding magnetic-field–based neuromodulation. A growing body of mechanistic research suggests that magnetic stimulation can influence nociceptive processing through both central and peripheral pathways. Experimental human work has shown that magnetic stimulation–induced analgesia depends on endogenous opioid mechanisms, as high-frequency rTMS produces frequency-dependent pain reduction that is attenuated when opioid-related pathways are pharmacologically blocked [40]. Beyond central neuromodulation, pulsed electromagnetic fields have been shown to exert significant peripheral biological effects, including reductions in inflammatory mediators, improvements in microcirculation, and enhancements in cellular metabolism and ATP synthesis, all of which may contribute to decreased peripheral nociceptor sensitization [16,41]. Collectively, these findings support the hypothesis that, rather than acting as a passive physical modality, FMS may function as an active neuromodulatory stimulus capable of engaging both opioid-dependent and metabolic pathways to produce analgesic effects when combined with manual therapy. These proposed mechanisms should be interpreted as plausible hypotheses derived from indirect experimental and clinical evidence, rather than as confirmed mechanisms demonstrated by the present trial.

A growing body of mechanistic research suggests that electromagnetic stimulation exerts complementary analgesic effects that align with and potentially amplify the responses produced by manual therapy and neurodynamic techniques. Experimental and clinical evidence suggests that pulsed electromagnetic fields can reduce nociceptive gain, attenuate neuroinflammatory activity, enhance microcirculation, and promote ATP-dependent cellular recovery processes relevant to pain modulation [16,41]. In parallel, human neuroimaging studies have shown that magnetic stimulation can activate endogenous μ-opioid networks, providing a plausible central mechanism for enhanced descending inhibition and modulation of thalamo-cortical pain processing [40]. When these neurophysiological effects occur simultaneously with the mechanoreceptor-driven afferent input generated by manual and neurodynamic mobilization, the resulting multimodal sensory integration may contribute to a more robust neuromodulatory response than either intervention alone. This interpretation should be regarded as a plausible hypothesis rather than a confirmed synergistic mechanism.

Importantly, the present protocol applied magnetic stimulation during movement, rather than as a passive modality delivered in isolation. Mobilization occurring within an active electromagnetic field may enhance neural tissue viscoelasticity, may improve intraneural microcirculation, and may reduce axoplasmic flow restriction—mechanisms previously described in neurodynamic literature [42]. At the same time, the magnetic field may augment the mechanical desensitization produced by neurodynamic mobilization. Mechanical tension–slack cycles are known to influence neural mechanosensitivity through effects on intraneural circulation, axoplasmic transport, and mechanoreceptor loading [42]. When this input is delivered concurrently with frequency-modulated magnetic stimulation, additional neuromodulatory effects may occur, including improved microcirculation, attenuation of inflammatory activity, and modulation of nociceptive transmission [16,41]. Experimental neuroimaging evidence also indicates that magnetic stimulation can activate endogenous μ-opioid networks, providing a plausible basis for enhanced descending inhibition during combined treatment [40]. Taken together, these mechanisms suggest that the simultaneous application of mechanical and electromagnetic stimulation may create a multimodal sensory input that could contribute to a more effective neuromodulatory response; however, this hypothesis requires further investigation.

Collectively, these mechanisms may offer a plausible explanation for the greater improvements observed in pain, disability, neuropathic symptoms, and SLR mechanics in the FMS-enhanced treatment arm. Rather than functioning solely as a passive physical agent, FMS may be interpreted as a neuromodulatory facilitator that could potentially augment the effects of manual therapy by delivering concurrent mechanical and electromagnetic stimulation to the neuromusculoskeletal system.

The magnitude of improvements observed in this trial translates into meaningful functional benefits for patients with lumbar disk herniation and radiculopathy. The reductions in both lumbar and leg pain in the FMS group consistently exceeded established MCID thresholds [7,21,22], indicating that the analgesic response was not only statistically significant but also perceptible and relevant to everyday functioning. The substantial decrease in S-LANSS scores is particularly noteworthy: only participants receiving the combined intervention improved beyond the diagnostic threshold of ≥12 [7,26], suggesting a potentially important shift away from a neuropathic pain phenotype. This shift is clinically relevant, given that neuropathic mechanisms are generally associated with greater pain severity, poorer prognosis, and reduced responsiveness to conservative care. However, these interpretations should be considered preliminary and warrant confirmation in future trials with larger samples.

Equally important, although this finding should be interpreted with caution, is the improvement observed in SLR range, which may reflect a reduction in neural mechanosensitivity. The 20.85° increase achieved in the FMS group exceeded both the minimally detectable change (7–8°) [29] and the threshold for clinically important improvement (≥16°) [30]. Such changes are consistent with potential improvements in neural mobility, reduced intraneural pressure, and increased tolerance to neurodynamic load—factors that have been associated with functional recovery in radiculopathy. Improvements in disability (RMDQ) also surpassed the MCID threshold in the FMS group [21], suggesting that pain reduction was accompanied by functional gains in daily activities, mobility, and overall capacity. However, these findings should be confirmed in future studies with larger samples.

Taken together, these findings suggest that integrating FMS with manual therapy may be associated with a more comprehensive therapeutic effect that influences multiple domains of patient health, including nociceptive and neuropathic pain, neural mechanosensitivity, and functional disability. This multidimensional pattern of improvement supports the hypothesis that incorporating neuromodulatory electromagnetic stimulation into neurodynamic and manual therapy programs could potentially offer added clinical value for individuals with chronic lumbar radiculopathy.

The present findings may contribute to a broader conceptual shift in musculoskeletal rehabilitation by questioning the traditional view of physical agents as inherently passive modalities. Rather than acting solely as passive inputs, electromagnetic stimulation—when integrated with movement-based and manual techniques— may be conceptualized as an active neuromodulatory adjunct capable of influencing sensory processing, reducing neural sensitivity, and potentially facilitating functional recovery. This interpretation is aligned with emerging evidence from multimodal intervention studies, including recent work suggesting that combining physical agents with manual therapy may be associated with additive or synergistic effects on pain modulation and motor function [36,38,39].

From a neurophysiological standpoint, the combination of mechanical mobilization and frequency-modulated magnetic stimulation may provide a multisensory input to the central nervous system, potentially engaging mechanoreceptive, proprioceptive, and electromagnetic pathways simultaneously [16,41,42]. Such multimodal sensory input may contribute to sensorimotor integration, may support cortical reorganization, and may enhance descending inhibitory mechanisms [40,41] —effects that are less likely to occur when physical agents are applied in isolation or in a fully passive context. Accordingly, physical agents such as FMS might be interpreted not merely as preparatory adjuncts, but as potentially active components within a dynamic, movement-based therapeutic environment [16,41]. The present results are consistent with this perspective, suggesting that the clinical value of physical modalities may be optimized when they are embedded within functional, mechanically meaningful interventions that leverage their neuromodulatory potential.

This randomized controlled trial incorporates several methodological features that enhance the credibility and clinical relevance of the findings. First, the study employed a two-timepoint design with standardized baseline and post-intervention assessments, allowing for a structured evaluation of short-term treatment effects. Second, outcome assessment was conducted by an independent evaluator who was blinded to group allocation, thereby reducing the risk of detection bias. Treatment delivery followed a standardized protocol supported by therapist training and fidelity monitoring, which contributed to maintaining consistency in how the interventions were applied. Adherence was high, with all participants completing the treatment protocol and both assessment timepoints. The use of validated and widely accepted outcome measures—including the NPRS, RMDQ, S-LANSS, and SLR—further supports the interpretability of the results. Finally, the effect sizes observed across the primary outcomes were substantial, suggesting that the improvements detected are likely to be clinically meaningful and providing additional support for the methodological rigor of the study.

The present findings may have meaningful implications for clinical practice, particularly for physiotherapists and manual therapy practitioners treating individuals with lumbar disk herniation and radiculopathy. The results suggest that integrating frequency-modulated magnetic stimulation with conventional manual therapy and neurodynamic mobilization could potentially provide additional benefits in pain reduction, neural mechanosensitivity, and functional recovery compared with manual therapy alone. Clinically, FMS may be applied during mobilization procedures—rather than as a separate passive modality—allowing mechanical and electromagnetic stimuli to interact within the same treatment context. This approach may be particularly helpful for patients presenting with higher levels of radicular pain, restricted SLR range, or neuropathic pain features, who often respond more favorably to multimodal neuromodulatory strategies. From a practical standpoint, FMS can be delivered along the course of the sciatic nerve during mobilization and progressed according to symptom irritability, in a manner that parallels graded loading principles familiar to manual therapists. Overall, these findings encourage clinicians to consider the potential value of using physical agents such as FMS in a more active and integrated manner within functional movement-based interventions. From an implementation perspective, however, the integration of functional magnetic stimulation into routine clinical practice may be influenced by practical considerations such as equipment availability, associated costs, and the need for clinician training. These factors were beyond the scope of the present trial but may affect the feasibility and scalability of movement-integrated FMS protocols across different clinical settings.

Although the present study provides preliminary evidence supporting the short-term benefits of combining FMS with manual therapy, several questions remain. Future research should examine the durability of these effects through medium- and long-term follow-up, as changes in pain and neural mechanosensitivity may evolve over time. In particular, longitudinal designs incorporating multiple follow-up time points (e.g., 3, 6, and 12 months) would be valuable to determine whether the observed clinical improvements are sustained, diminish over time, or require maintenance or booster interventions in patients with chronic LDHR, as previous research in lumbar radiculopathy has predominantly reported short-term outcomes with limited long-term follow-up [8,10]. Mechanistic studies using neurophysiological tools—such as quantitative sensory testing (QST), electroencephalography (EEG), functional imaging, or microneurography—could help clarify how electromagnetic fields interact with mechanical mobilization at both peripheral and central levels. Further exploration of dose–response characteristics, including optimal frequency ranges, intensity, session duration, and timing of application relative to movement, may also help refine treatment protocols. Comparative trials evaluating FMS against other commonly used physical agents (e.g., laser therapy, TENS or TECAR therapy) would be valuable for determining whether electromagnetic stimulation offers distinct neuromodulatory advantages. Finally, future studies may benefit from examining patient subgroups to identify predictors of response and to guide more targeted multimodal interventions.

In summary, this randomized controlled trial suggests that integrating frequency-modulated magnetic stimulation with manual therapy and neurodynamic mobilization may provide additional clinical benefits for patients with lumbar disk herniation and radiculopathy compared with manual therapy alone. The combined intervention was associated with meaningful reductions in pain, neuropathic features, and neural mechanosensitivity, along with notable improvements in functional outcomes. These findings support the hypothesis that physical agents such as FMS may be conceptualized as active neuromodulatory adjuncts rather than passive modalities and highlight the potential value of multimodal, movement-integrated approaches within contemporary musculoskeletal rehabilitation.

This study has several limitations that should be acknowledged when interpreting the findings. First, the sample size, although adequately powered for the primary analyses, was relatively small and limits the generalizability of the results to broader clinical populations. Second, the study included only individuals with chronic LDHR; therefore, the findings may not extend to patients in the acute phase or those with different etiologies of sciatic pain. Third, the follow-up period was limited to three weeks, capturing only short-term responses to treatment. Without long-term tracking, it remains unclear whether the observed improvements persist, diminish, or further progress over time. Accordingly, the short-term improvements observed in this trial should not be assumed to translate into sustained long-term benefits, particularly in a chronic condition such as LDHR. Furthermore, the interpretation of clinical relevance was based on established MCID thresholds derived primarily from broader low back pain populations, as condition-specific MCID values for patients with LDHR are not well defined. Although these thresholds are widely used and represent the best available reference, they may not fully capture what constitutes a clinically meaningful change in this specific population and should therefore be interpreted with appropriate caution. In addition, the study did not include a sham magnetic stimulation control condition, and neither participants nor therapists were blinded to treatment allocation. As a result, placebo effects and expectancy-related influences cannot be fully excluded, particularly given the novelty of the intervention. Additionally, although outcome assessment was conducted by a blinded assessor, the primary outcomes relied on validated self-report instruments, which inherently involve subjective patient perception and may be influenced by expectancy or contextual factors. Moreover, because FMS was applied concurrently with neurodynamic manual therapy techniques, the present study design does not allow isolation of the independent effects of each modality. Potential interaction effects arising from the simultaneous mechanical and electromagnetic stimulation cannot, therefore, be disentangled and should be considered when interpreting the results. Moreover, concurrent use of analgesic medication, changes in physical activity levels, or other self-managed interventions during the treatment period were not systematically monitored, and their potential influence on outcomes cannot be fully excluded. Finally, while the study was rigorously controlled, future research could incorporate additional objective biomarkers—such as neurophysiological or imaging measures—to more comprehensively characterize the mechanisms underlying the observed clinical effects.

## 5. Conclusions

In summary, this randomized controlled trial suggests that combining manual therapy with frequency-modulated magnetic stimulation may provide potential additional short-term benefits for individuals with chronic lumbar disk herniation with radiculopathy compared with manual therapy alone. Participants receiving the combined intervention demonstrated greater improvements in pain intensity, neuropathic pain features, disability, and straight-leg-raise range, with several changes exceeding established thresholds for clinical relevance. Notably, reductions in S-LANSS scores surpassed the diagnostic cutoff for neuropathic pain only within the FMS group, indicating a potentially meaningful shift in symptom profile.

These findings support preliminary evidence that integrating FMS with manual therapy may yield additive therapeutic effects, potentially enhancing neuromodulatory, analgesic, and mechanosensitive mechanisms. Delivering mechanical mobilization within an electromagnetic field may represent a promising, yet exploratory, multimodal approach to rehabilitation for patients with lumbosacral radiculopathy; however, this interpretation should be considered preliminary.

Given the short intervention duration and the absence of long-term follow-up, further research with larger samples, extended monitoring, and mechanistic neurophysiological assessments is needed to determine the durability of these effects and to clarify the underlying mechanisms. Overall, the present results suggest the potential value of an FMS-enhanced manual therapy approach, while highlighting the need for additional studies to confirm and extend these findings.

## Figures and Tables

**Table 1:** Components of manual therapy protocol.

**1. Spinal Mobilization With Leg Movement (SMWLM)** For SMWLM, participants were positioned in side-lying, with the symptomatic leg uppermost, the hip flexed to approximately 90°, and a foam roll placed beneath the thigh to unload the limb and stabilize the pelvis. The therapist applied a sustained lateral or postero-anterior glide over the symptomatic lumbar segment (typically L4/5 or L5/S1) using both thumbs, while the participant performed active knee extension in a pain-free or acceptable range. Three sets of 6–10 repetitions per affected level were delivered, allowing 30–60 s rest between sets. Glide direction, segmental level, and amplitude were continuously titrated according to symptom response, following established Mulligan principles [6,31].
**2. Neurodynamic Mobilization (Sliders-Tensioners)** Neurodynamic techniques targeted the sciatic nerve and were delivered in sitting, in accordance with current clinical standards. **Weeks 1–2: Symptom-limited “Sliders”** During the first two weeks, symptom-modulated sliders were performed with the participant seated, the knee extended to a position just short of symptom onset (typically ~10–15° before the elicited angle), and rhythmic oscillations of the ankle between dorsiflexion and plantarflexion were performed at 0.5–1 Hz. To maintain the slider mechanism, cervical movement was coupled with the distal oscillation—for example, slight cervical extension during ankle dorsiflexion and flexion during plantarflexion—always within a comfortable, non-provocative range. Three bouts of 60 s were applied, each followed by 60 s of rest. Movements were restricted to a non-provocative range, maintaining symptoms ≤ 3/10 and avoiding next-day exacerbation. **Week 3: Progression to “Tensioners”** In the third week—and only when irritability was low (pain ≤ 3/10 during execution, and no >24 h flare)—mobilization progressed to brief tensioners, also in sitting. From the symptom-onset angle, the participant performed 2–3 s end-range dorsiflexion holds, for 3 sets of 10–12 repetitions. All dosing followed a symptom-guided titration principle, avoiding sustained increases in radicular pain [7,32].
**Home Program** A brief daily home program of seated sciatic sliders (2–3 times per day; 3 sets × 10 repetitions) within a pain-acceptable range was prescribed. Participants were given a simple green–amber–red self-regulation rule (traffic-light model) to adjust dosage according to next-day symptom behavior.

**Table 2:** Baseline demographic characteristics of the participants. Quantitative variables are reported as mean ± SD and categorical variables as n (%). Group differences were assessed using independent-samples t-tests (a) and chi-square tests (b).

Demographic Variable	Manual Therapy and FMS Group (n = 20)	Manual Therapy Group (n = 20)	*p*-Value (Between Groups)
Age (years) (Mean ± SD)	45.15 ± 11.05	44.80 ± 11.19	0.92 ^a^
Sex (Male/Female)	20.0% (n = 4) Male 80.0% (n = 16) Female	15.0% (n = 3) Male 85.0% (n = 17) Female	0.67 ^b^
Affected side (Right/Left)	65.0% (n = 13) Right 35.0% (n = 7) Left	50.0% (n = 10) Right 50.0% (n = 10) Left	0.33 ^b^
Level of disk herniation (L4–L5/L5–S1)	70.0% (n = 14) L4–L5 30.0% (n = 6) L5–S1	75.0% (n = 15) L4–L5 25.0% (n = 5) L5–S1	0.72 ^b^
Symptoms duration (months) (Mean ± SD)	9.65 ± 2.62	9.15 ± 2.23	0.52 ^a^
BMI (kg/m^2^) (Mean ± SD)	25.31 ± 2.31	25.38 ± 1.63	0.91 ^a^

Mean ± SD = Mean value ± standard deviation. ^a^ Independent samples *t*-test. ^b^ Chi-square test. NPRS–Lumbar Pain results.

**Table 3:** Mean (SD) values of outcome measures for the Manual Therapy + FMS and Manual Therapy Only groups at baseline and post-intervention, including *p*-values.

Outcome	Baseline (Mean ± SD)	Week 3 (Mean ± SD)	Mean Difference	Cohen’s d	95% CI	Time Effect F(*p*, η^2^p)	Interaction *p*-Value
NPRS–Lumbar Pain (score)							
Manual Therapy + FMS	5.80 ± 1.54	2.05 ± 1.39	−3.75	2.55	(−2.18, −0.22) *	F = 130.68, *p* < 0.001, η^2^p = 0.775	0.005
Manual Therapy Only	5.45 ± 1.70	3.25 ± 1.65	−2.20	1.31			
NPRS–Leg Pain (score)							
Manual Therapy + FMS	6.40 ± 2.14	2.40 ± 1.50	−4.00	2.35	(−2.52, −0.38) *	F = 893.78, *p* < 0.001, η^2^p = 0.959	< 0.001
Manual Therapy Only	5.70 ± 1.78	3.85 ± 1.81	−1.85	1.01			
RMDQ (score)							
Manual Therapy + FMS	14.00 ± 4.18	4.05 ± 3.15	−9.95	2.62	(−5.64, −1.06) *	F = 511.89, *p* < 0.001, η^2^p = 0.931	< 0.001
Manual Therapy Only	13.20 ± 4.15	7.40 ± 3.97	−5.80	1.47			
S-LANSS (score)							
Manual Therapy + FMS	14.65 ± 4.20	4.60 ± 2.78	−10.05	2.35	(−4.66, −0.24) *	F = 848.47, *p* < 0.001, η^2^p = 0.957	0.001
Manual Therapy Only	14.90 ± 4.53	7.05 ± 4.02	−7.85	1.75			
SLR (degrees)							
Manual Therapy + FMS	45.90 ± 5.62	66.75 ± 7.58	+20.85	2.92	(2.70°, 11.50°) *	F = 1394.13, *p* < 0.001, η^2^p = 0.973	< 0.001
Manual Therapy Only	47.75 ± 6.19	59.65 ± 6.10	+11.90	1.85			

Note. CI values correspond to between-group differences at Week 3, extracted from parameter estimates. All effect sizes (Cohen’s d) represent within-group improvement using baseline → Week 3 SD_pooled. Asterisks (*) denote statistically significant between-group differences.

## Data Availability

The data that support the findings of this study are openly available in Zenodo at https://doi.org/10.5281/zenodo.17936953.

## Supplementary Materials

The following supporting information can be downloaded at https://www.mdpi.com/article/10.3390/medicina62020249/s1. CONSORT checklist (PDF).
